# Food Insecurity in Older Adults: Gerontology Students and RSVP a Partnership that Works

**DOI:** 10.1093/geroni/igab046.3056

**Published:** 2021-12-17

**Authors:** Phyllis Greenberg, Jessica VanderWerf

**Affiliations:** 1 Saint Cloud State University, St. Cloud, Minnesota, United States; 2 St. Cloud State University, Saint Cloud State University, Minnesota, United States

## Abstract

A gerontology course related to policies /programs each year researches, develops and designs a service-learning project related to an issue/concern for older adults and their quality of life. Students wanted to work with vulnerable older adults and after research and discussion decided on tackling the issue of food insecurity in older adults. Food insecurity is a growing issue for older adults which has been exasperated by COVID-19. According to Meals on Wheels America (2020) there has been a 22% increase in the number of older adults needing food assistance. In addition, while the need for food banks has increased donations have declined (Next Avenue, 2020). Students partnered with RSVP, which had previously conducted a food donation project.. Students took on the responsibility for advertising, soliciting grocery stores to allow us to set up and engage shoppers in purchasing items for the project. In addition, they reached out to the university community and set up food donation stations. RSVP sent out emails to their constituents to encourage them to volunteer and do their shopping on the date of the project. Students were paired with RSVP volunteers at two stores and provided shopping lists and information about food insecurity in older adults to shoppers. Students collected 566 pounds of food. The food was distributed equally between Catholic Charities, which has a senior shopping program and the Somali Elder Community. Students sorted the food by categories and removed any foods with pork/gelatin products for the Somali Community.

